# Comparison of the cardiovascular system, clinical condition, and laboratory results in COVID-19 patients with and without vitamin D insufficiency

**DOI:** 10.1186/s12879-022-07438-8

**Published:** 2022-05-07

**Authors:** Erfan Kazemi, Ali Mansursamaei, Marzieh Rohani-Rasaf, Hossein Sheibani

**Affiliations:** 1 Student Research Committee, School of Medicine, Shahroud University of Medical Sciences, Shahroud, Iran; 2 Clinical Research Development Unit, Imam Hossein Hospital, Shahroud University of Medical Sciences, Imam Ave., Shahroud, 3616911151 Iran; 3 Department of Epidemiology, School of Public Health, Shahroud University of Medical Sciences, Shahroud, Iran

**Keywords:** Vitamin D, COVID-19, Morbidity, Mortality, Cardiovascular

## Abstract

**Background:**

Serum vitamin D levels may have a protective role against severe coronavirus disease 2019 (COVID-19). Studies have shown that deficiency in vitamin D may be a significant risk factor for poor outcomes. This study aims to compare the outcome and clinical condition of patients diagnosed with COVID-19 infection considering serum vitamin D levels.

**Methods:**

In this cross-sectional study, 202 COVID-19 patients without known cardiovascular disease (reduced ejection fraction, uncontrolled arrhythmia, pericardial effusion, cardiac block, valvular disease, or hypertension) were included. Patients were divided into three groups of insufficient (< 30 ng/mL), normal (30 to 50 ng/mL), and high (> 50 ng/mL) serum vitamin D levels. Clinical outcome was defined as severe if invasive respiratory intervention and ICU admission was required.

**Results:**

The patients were divided into three groups based on their vitamin D level: 127 cases in the insufficient vitamin D group, 53 cases in the normal vitamin D group, and 22 cases in the high vitamin D group. The mean age of the population study was 56 years. Thirty-four patients had severe clinical outcomes. The distribution of this group was as follows: 21 patients in the insufficient vitamin D group (16.5%), eight patients in the normal vitamin D group (15.1%), and five patients in the high vitamin D group (22.7%); P = 0.74. No significant differences were found between the groups in terms of mortality rate (P = 0.46). Moreover, the mean of leukocytes (mean ± SD = 6873.5 ± 4236.2), ESR (mean ± SD = 38.42 ± 26.7), and CPK-MB (mean ± SD = 63 ± 140.7) were higher in the insufficient vitamin D group, but it was not statistically significant (P > 0.05).

**Conclusion:**

The finding of the present study showed that vitamin D could not make a significant difference in cardiovascular systems, laboratory results, and severity of the disease in COVID-19 patients.

## Background

Coronavirus disease 2019 (COVID-19) is a contagious infectious disease caused by severe acute respiratory coronavirus 2 (SARS-CoV-2) [1, 2]. The COVID-19 pandemic impressed humanity life in several sectors such as education, health, and politic [3]. According to the World Health Organization (WHO) reports, nearly 269 million confirmed cases had been infected with this virus, with 5.3million deaths globally as of 12 December 2021[4]. This disease has risk factors including older age, smoking, underlying conditions like diabetes, hypertension, heart disease, chronic lung diseases, and immunosuppression [2, 5, 6].

Recently, a genomic-guided tracing of SARS-CoV-2 targets in human cells spotted that vitamin D is among molecules with potential infection alleviation patterns through gene expression [7]. Vitamin D is a key hormone that contributes to the modulation of the innate and acquired immune system by decreasing pro-inflammatory cytokines (like interferon-gamma and interleukin-2) and increasing concentrations of anti-inflammatory cytokines. Furthermore, it may limit the infection by expressing genes involved in the intracellular destruction of pathogens and lowering the viral replication rate [1, 2, 8–12]. It also modifies the renin-angiotensin pathway and down-regulates angiotensin-converting enzyme (ACE-2), with which the virus can enter the target cells [13, 14]. Therefore, vitamin D might help treat COVID-19 by preventing the cytokine storm and subsequent acute respiratory distress syndrome (ARDS).

Some studies reported that vitamin D deficiency is a risk factor for infection with COVID-19 disease [8, 15, 16]. This finding was confirmed in a meta-analysis of 361,934 subjects [10]. Several clinical trials and observational studies demonstrated the association between vitamin D deficiency with mortality in COVID-19 patients and the beneficial effect of vitamin D supplementation on the outcome [1, 6, 7, 12, 14, 16–19]. On the other side, the mentioned beneficial effects of vitamin D in COVID-19 have not been indicated in other studies [2, 20]. To illustrate, in a cohort study with 105 patients, vitamin D status prognosticated COVID-19 infection in univariate Analysis but not after adjustment for covariates. Considering that the association of vitamin D with the risk of COVID-19 disease may be a spurious relationship caused by the confounding effects of comorbidities, associations found in these studies may be, in fact, affected by the potential effects of critical illness on total vitamin D levels [21]. In other words, vitamin D deficiency in many diseases may be due to the negative effect of underlying diseases and related inflammatory background on vitamin D metabolism [19].

It has been reported that vitamin D has extra-skeletal effects, especially on the cardiovascular system. In addition, vitamin D receptor is identified in almost all tissues, including the heart and the blood vessels [22–24]. Some clinical and observational evidence showed the possible association of low levels of 25-hydroxy-vitamin D (25OHD, the active form of vitamin D) with a higher risk of cardiovascular diseases, including hypertension, coronary artery disease, ischemic heart disease, heart failure, and stroke [22, 23]. Cardiovascular diseases are one of the most important risk factors for mortality and morbidity in COVID-19 [25, 26]. Therefore, we decided to compare clinical outcomes, in particular cardiovascular outcomes, among COVID-19 patients based on their serum vitamin D levels.

## Materials and methods

### Patient population and study design

In this cross-sectional study, 202 COVID-19 patients who were hospitalized in Imam Hossein Hospital, Shahroud, Iran, from late February to late June of 2020 were included. Patients with a history of reduced left ventricular ejection fraction (LVEF) of less than 55%, uncontrolled arrhythmia, pericardial effusion (moderate to massive), valvular disease (moderate to severe), or uncontrol or resistant hypertension were not included. COVID-19 identified if one RT-PCR test or CT-scan becomes positive in patients. RT- nasopharyngeal samples were used for the PCR test (Sansure Co., China). If the viral genome [RdRP and Nucleocapsid (N) genes] were detected, the test was considered a positive result. The RT-PCR was repeated if the primary result was suspicious. Additionally, the cardiovascular history of patients was documented through their medical records and medication history. Serum vitamin D levels were assayed during hospitalization using the Vidas kit (Biomerieux Co., France). The patients were divided into three groups insufficient (< 30 ng/mL), normal (30 to 50 ng/mL), and high (> 50 ng/mL) vitamin D levels. Transthoracic echocardiography was done by 10 years experienced cardiologist for all the patients using General Electric (GE) set model VIVID S6. ECG had been taken several times during hospitalization. Then, if we could find any change such as ST-segment changes (inverted T wave, T-flat, ST-depression, ST-segment elevation), atrioventricular (AV) blocks, arrhythmia in even one of the ECGs, the variable would be considered positive for that patient. Laboratory results and clinical signs were measured one time. Variables that were measured were clinical condition, LVEF, arrhythmia, ST-segment changes, blood pressure, block arrhythmia, intubation, hospitalization duration, comorbidity, in-hospital death, pulse rate (normal, abnormal), respiratory rate, O_2_ saturation, gender, pericardial effusion, erythrocyte sedimentation rate (ESR), platelet counts, leukocyte count, creatinine, fast blood sugar (FBS), blood sugar (BS), body mass index (BMI), lactate dehydrogenase (LDH), CPK-MB, C-reactive protein (CRP), and troponin.

### Definitions

The cutoff point for sufficient vitamin D level was 30 ng/mL [27]. Clinical outcome was defined as severe if invasive respiratory intervention and ICU admission was required. Otherwise, the clinical outcome was categorized as a non-severe outcome. High blood pressure defines even if one of these conditions happens: systolic blood pressure ≥ 130 mmHg or diastolic pressure ≥ 80 mmHg [28]. In addition, patients with acute respiratory disease had at least one of the below conditions admitted in hospital-based on the physician’s opinion: (1) respiratory rate > 30/min (2) PO_2_ < 93% without any oxygen supplement (3) pulmonary infiltration in chest X-ray caused by COVID-19 (4) medical judgment of an expert physician.

In case of any of the following criteria, the admission of the patient to the ICU was indicated: threatened airway, respiratory arrests, respiratory rate ≥ 40 or ≤ 8 breaths/min, oxygen saturation < 90% on ≥ 50% oxygen, cardiac arrests, pulse rate < 40 or > 140 beats/min, systolic blood pressure < 90 mmHg, sudden fall in the level of consciousness (fall in Glasgow Coma Score > 2 points), repeated or prolonged seizures, rising arterial carbon dioxide tension with respiratory acidosis, and any patient giving cause for concern.

### Ethics

The current study was approved by the medical ethics committee of Shahroud University of Medical Sciences, Shahroud, Iran (IR.SHMU.REC.1399.014). The study was performed according to the guidelines of the last version of the Declaration of Helsinki.

### Statistical analysis

The chi-square or Fisher exact tests were used for comparing the qualitative variables among the categorical groups of vitamin D. Independent samples t-test, and ANOVA was run to compare the means of the quantitative variables between groups. Pearson correlation test was used to determine the correlation between serum vitamin D levels and clinical or laboratory variables. The analyses were done using SPSS software (ver. 16.0). P values of less than 5% were considered statistically significant.

## Results

Overall, 202 patients were included. The mean (± SD) age of patients was 56.8 (± 14.3) years; 90 patients (44%) were older than 60 years. There were 107 male cases.

One hundred nine patients (53.9%) had at least one comorbid condition. Among all patients, 54% had at least a history of a chronic disorder; 2 patients had a malignant condition, 59 patients had diabetes, 2 patients had HIV infection, 63 patients had cardiovascular diseases, 11 patients had asthma, 9 patients had chronic kidney disease, 18 patients had renal disease, and 14 patients had cerebrovascular diseases.

Eight patients (4%) died of COVID-19 infection. The clinical and cardiovascular outcomes of the patients are summarized in Table 1. Although the prevalence of COVID-19 patients with severe clinical outcomes in the high vitamin D group (22.7%) was higher than in other groups, it was not meaningful (p = 0.74). The patients need to intubation [number = 4 (18.2%), P = 0.34] was higher in high vitamin group. However, the mortality rate [number = 7 (5.4%), P = 0.46] was higher in the patients with insufficient vitamin D levels due to the Fischer test, but it was not significant.

**Table 1:** Comparison of cardiovascular variables and laboratory test among three groups of COVID-19 patients with low, normal, and high serum vitamin D levels

	Insufficient vitamin D (N = 127)	Normal vitamin D (N = 53)	High vitamin D (N = 22)	P value
Reduced ventricular ejection fraction EF decreased (< 55%)	23 (18%)	17 (32%)	5 (22.7%)	0.12
Arrhythmia	4 (3%)	5 (9.4%)	3 (13.6%)	0.09
ST segment changes	93 (73.2%)	39 (73.6%)	17 (77.3%)	0.58
Pericardial effusion	25 (19.7%)	14 (26.4%)	2 (9%)	0.53
Positive troponin	4 (3%)	3 (5.6%)	–	0.52
Block	17 (13.4%)	4 (7.5%)	6 (27.3%)	0.06
Severe clinical outcome	21 (16.5%)	8 (15.1%)	5 (22.7%)	0.74
ESR	38.42 ± 26.7	35.5 ± 28.7	35.6 ± 21.5	0.8
Platelet count	209,876 ± 87,504	17,880 ± 60,330.4	192,650 ± 66,724.3	0.06
Leukocyte count	6873.5 ± 4236.2	5764 ± 2867.8	5600 ± 2145.7	0.1
Creatinine	1.1 ± 0.9	1.3 ± 1.3	1 ± 0.3	0.5
Lymphocyte	1745.1 ± 518	1623.1 ± 365.82	1524.3 ± 354.8	0.4
LDH	498.6 ± 248.6	834.4 ± 1957.7	435.6 ± 150	0.2
CPK-MB	63 ± 140.7	35.3 ± 27.4	22.6 ± 12.9	0.7
LVEF	53.2 ± 73.4.6	51.3 ± 8.3	54.6 ±	0.2
Respiratory rate	10.94 ± 8.9	10.67 ± 9.1	12 ± 8.6	0.9
O_2_ saturation	90.64 ± 7.3	90.21 ± 8.5	93.17 ± 7.3	0.5
Duration of hospitalization	7.4 ± 6.7	7.3 ± 5.3	8.1 ± 7	0.9

*ESR* erythrocyte sedimentation rate, *LDH* lactate dehydrogenase, *CPK-MB* creatine phosphokinase-MB, *LVEF* left ventricular ejection fraction

Table 1 shows the comparison of the laboratory variables between the three groups. As observed, no statistically significant difference was detected between the three groups of patients with insufficient, normal, and high serum vitamin D levels. The mean of FBS in mean levels of vitamin D were also compared in the subsets of the studied variables (Table 2). Patients with normal and high blood pressure had almost equal levels of vitamin D. Patients with severe clinical conditions had insufficient concentrations of vitamin D, but the difference did not reach a significant level.

**Table 2:** Comparison of serum vitamin D between subset of patients according to categorical variables

Characters	Serum vitamin D level (mean ± SD)	P value	Mean difference	Confidence interval (CI)
Gender				
Male	28.3 ± 19.6	0.85	0.49	(− 4.8, 5.8)
Female	27.9 ± 18.6			
Decreased LVEF				
Yes	27.1 ± 17.6	0.15	− 0.4.61	(− 10.9, 1.7)
No	31.7 ± 23.2			
Clinical condition				
Severe	27.2 ± 18	0.08	− 6.56	(− 14, 0.9)
Non-severe	33.7 ± 23.9			
Pericardial effusion				
Yes	29.1 ± 26.6	0.4	4.39	(− 7, 15.8)
No	33.5 ± 26.6			
CRP				
Positive	27.2 ± 17.5	0.5	− 2.11	(− 9.2, 5)
Negative	25.1 ± 12.4			
ST segment changes				
Yes	27.9 ± 18.3	0.2	4.90	− 2.7, 12.4)
No	32.8 ± 24.5			
Blocks				
Yes	34.3 ± 25.7	0.17	− 7.10	(− 17.6, 3.3)
No	27.1 ± 17.7			
Arrhythmia				
Yes	44.2 ± 18.1	0.09	− 16.6	(− 36.5, 3.3)
No	27.7 ± 18.1			
Blood pressure				
Normal	28.9 ± 21.9	0.6	1.5	(− 4, 7.1)
Hypertension	27.4 ± 17.3			

*LVEF* left ventricular ejection fraction, *CRP* C-reactive protein

Table 3 presents the correlation between serum vitamin D concentration and clinical/laboratory variables. As observed, no significant correlation was detected between vitamin D and other variables.

**Table 3:** Correlation between serum vitamin D level and clinical/laboratory variables

Variables	Serum vitamin D level	
	Pearson correlation coefficient	P value
Respiratory rate	0.04	0.7
O_2_ saturation	0.11	0.2
ESR	0.01	0.8
CPK-MB	− 0.13	0.4
Leukocyte count	− 0.12	0.08
Creatinine	− 0.03	0.6
LDH	− 0.01	0.8
Lymphocyte count	0.03	0.6
Platelet count	− 0.12	0.09
LVEF	− 0.01	0.8
BS	− 0.33	0.7
FBS	− 0.9	0.3

*ESR* erythrocyte sedimentation rate, *CPK-MB* creatine phosphokinase-MB, *CPK-M*: creatine phosphokinase-MB, *LDH* lactate dehydrogenase, *LVEF* left ventricular ejection fraction, *BS* blood sugar, *FBS* fast blood sugar

## Discussion

Previous studies have shown the possible relationship between vitamin D status and respiratory infections [27, 29]. Since studies have demonstrated that a sufficient vitamin D level is associated with a better immune system function, researchers posed vitamin D might have a protective effect against COVID-19 [30, 31]. In addition, patients with severe COVID-19 may share some characteristics with those with insufficient vitamin D levels, such as pre-hospital malnutrition, liver or kidney dysfunction, older age, and a poor general health condition [32]. All these factors may affect the hospitalization rate, the likelihood of ICU admission, and the mortality rate [33]. In that way, some retrospective studies have been conducted to assess this association. Although a relationship has been found between vitamin D levels and COVID-19 as well as the clinical condition of the disease, the authors reported that further studies are required [34, 35]. In the present study, the prevalence of insufficient vitamin D was 69.2%, similar to the other studies [27, 35].

Pimentel et al. assessed 26 patients and showed that the mean CRP did not differ significantly between patients with low and normal vitamin D levels, while the mean lymphocyte count was higher in patients with low vitamin D levels [36]. Lakkireddy et al. conducted a randomized clinical trial and showed that vitamin D therapy significantly reduced inflammatory factors in patients receiving vitamin D (CRP: 81 ± 66 vs. 16 ± 42; P < 0.0001, LDH: 369 ± 159 vs. 274 ± 115; P < 0.0001). This study declared that vitamin D could reduce inflammatory factors without any side effects in COVID-19 patients [37]. However, a randomized clinical trial (RCT) showed that vitamin D did not make a significant difference in CRP level (p value = 0.5) [38].

Moreover, in the present study, the pooled mean of vitamin D in patients with positive CRP (mean ± SD: 27.2 ± 17.5) was higher than in patients with negative CRP (mean ± SD: 25.1 ± 12.4), though the difference was not statistically significant [p value = 0.5, CI = (− 9.2, 5)]. Also, the pooled mean of lymphocyte and leukocyte count was higher in patients with insufficient vitamin D levels, though not statistically significant.

Davoudi et al. studied 153 COVID-19 patients and found no significant difference between the groups in terms of the need for invasive ventilation [27]. A retrospective study in Austria on 148 patients reported that vitamin D levels did not differ significantly between various types of oxygen therapy [39]. The present study showed no significant difference in intubation with the level of vitamin D (P = 0.46).

Abrishami et al. argued that the mortality rate in vitamin D deficient patients was significantly higher than in patients with sufficient vitamin D levels (34.6% vs. 6.4%, P = 0.003). A deficient vitamin D level increased the hazard of mortality rate in an adjusted model (HR = 4.15, P = 0.04). The mean O_2_ saturation was lower in those who died (88) than in those who survived (90), though the difference was not significant (P = 0.11). The authors suggested that vitamin D might have a protective effect against the progression of COVID-19 to a severe form [6]. Al-Daghri et al. conducted a multi-center case–control study on 220 patients and described that COVID-19 patients had lower vitamin D than patients without COVID-19 (52.8 nmol/L vs. 64.5 nmol/L; P = 0.009) although COVID-19 patients had risk factors such as low HDL-c, diabetes mellitus, and old age [40]. In addition, some trials proved the protective effect of vitamin D against COVID-19, though their results were heterogeneous among their results, which may be possibly attributed to the following reasons: (1) administration of different doses of Vitamin D, and (2) dissimilar characteristics of participators [12].

In contrast, Butler-Laporte et al. compared 14,134 individuals with COVID-19 to 1,284,876 individuals without COVID-19. They did not find any relationship between elevated levels of vitamin D and COVID-19 susceptibility (odds ratio [OR] = 0.95; 95% CI 0.84, 1.08; P = 0.44), hospitalization (OR = 1.09; 95% CI 0.89, 1.33; P = 0.41), and severe disease (OR = 0.97; 95% CI 0.77, 1.22; P = 0.77). Their findings did not support the protective role of vitamin D in COVID-19 [41]. Moreover, another study on 502,624 participants showed that no association between vitamin D level and risk of COVID-19 [30].

A study in Iran showed mortality rate in the vitamin D sufficient patients was higher than in the vitamin D deficient patients (3.5% vs. 3.1%). Additionally, the mean hospitalization days in the vitamin D sufficient patients (6.36) was higher than the vitamin D deficient patients (6.25), though the difference was not statistically significant. In addition, univariate and multivariate linear regression analyses showed no association between vitamin D level with hospitalization duration and long-term complications [27]. Azadeh et al. demonstrated that the number of patients with insufficient or deficient vitamin D levels was not significant in both groups (dead/survived) (p value = 0.35) [42]. However, a single-center cohort study declared vitamin D deficiency increases the rate of in-hospital mortality after adjusting age and sex (OR = 1.73, CI = 1.11, 2.69) [43].

An observational study in Spain assessed 1549 patients. After adjusting gender and age, they concluded that low levels of vitamin D increase the risk of hospitalization and critical care, but not the mortality rate [35]. Additionally, another study in Iran showed that vitamin D insufficiency or deficiency was not significantly different between ICU and non-ICU patients [42]. A systematic review in Brazil studied all of the RCTs, which showed no association between the vitamin D group and placebo in terms of mortality, length of hospitalization stay, and duration of invasive ventilation. However, they included a few studies, and the dose of Vitamin D was different in 3 studies may confound their conclusion [44]. In contrast, two systematic reviews and meta-analyses proved a significant relationship between vitamin D deficiency and the severity of disease [45, 46]. However, these systematic reviews did not consider age and sex, included heterogeneous studies, and most parts of included studies were observational, avoiding causality conclusions. However, the rate of severe disease and the duration of hospitalization were higher in the patients with sufficient vitamin D levels than in patients with insufficient vitamin D levels [P = 0.74, P = 0.9 (8.1 ± 7 vs. 7.4)]. No significant differences were found between groups in terms of O_2_ saturation and mortality rate.

Some previous studies reported that low vitamin D levels might lead to coronary artery dysfunction, postinfarction complications, vasculitis, and adverse cardiovascular outcomes [47, 48]. Similarly, distinct research regarding the effectiveness of vitamin D on heart failure showed controversial results [49]. Christina et al. reported vitamin D can efficiently decrease the risk of ischemia (odds ratio = 0.934, confidence interval [0.882, 0.989]) [50]. On top of that, scientists have found that vitamin D deficiency may increase the risk of atrial fibrillation (AF), possibly since vitamin D can reduce inflammation by inducing interleukin 10 (IL-10) production and reducing the production of interferon-γ, and tumor necrosis factor-alpha (TNF-α), IL-6, and IL-12 [51], and vitamin D inhabitant the renin-angiotensin system (RAS) [52]. A review study in 2019 on the effect of vitamin D on atrial fibrillation reported that further basic and precise studies are required in this area [53].

On the other hand, researchers have shown the association between COVID-19 and cardiovascular complications, which posed the potential role of vitamin D levels and cardiovascular complications in COVID-19 patients. Zheng et al. demonstrated that COVID-19 could cause myocarditis through an uncertain specific mechanism [54]. Dinis et al. declared that COVID-19 patients with a critical condition had higher rates of arrhythmia. Moreover, atrial fibrillation and other supraventricular arrhythmia were the most prevalent amongst these patients. However, vitamin D levels were not related to increased cardiac mortality [55]. Additionally, a systematic review and meta-analysis demonstrated that QTc prolongation, ST-changes, and arrhythmia could be observed in COVID-19 patients. They also reported that cardiac arrhythmia was associated with poor prognosis [56]. Some studies reported an increased rate of myocardial infarction and heart failure due to vitamin D deficiency [57]. A systematic review and meta-analysis reported that COVID-19 might affect the cardiovascular system [58]. Consequently, in the present study, the cardiovascular factors were assessed in different levels of vitamin D. Findings of the present study showed no statistically significant difference between serum vitamin D levels in terms of blood pressure, arrhythmia, reduced LVEF, pericardial effusion, and abnormal ST-segment changes.

## Limitation

There were some limitations, including the small sample size and not gathering some factors such as smoking and socioeconomic status, which could affect the severity of COVID-19.

## Conclusion

There was no significant association between vitamin D level and clinical course and mortality rate in COVID-19. In addition, there was not even an association between vitamin D insufficient and duration of hospitalization. We advise performing some larger cohort studies and randomized clinical trials to assess this relationship in more detail.

## Data Availability

The datasets used and/or analyzed during the current study are available from the corresponding author on a reasonable request.

## Abbreviations

COVID-19: Coronavirus disease 2019
ICU: Intensive care unit
ARDS: Acute respiratory distress syndrome
LDH: Lactate dehydrogenase
CRP: C-reactive protein
LVEF: Left ventricular ejection fraction
ESR: Erythrocyte sedimentation rate
CPK-MB: Creatine phosphokinase-MB
